# Diverging prognostic effects of CD155 and CD73 expressions in locally advanced triple-negative breast cancer

**DOI:** 10.3389/fonc.2023.1165257

**Published:** 2023-07-13

**Authors:** Neslihan Cabioglu, Aysel Bayram, Selman Emiroglu, Semen Onder, Huseyin Karatay, Gizem Oner, Mustafa Tukenmez, Mahmut Muslumanoglu, Abdullah Igci, Adnan Aydiner, Pinar Saip, Ekrem Yavuz, Vahit Ozmen

**Affiliations:** ^1^ Department of General Surgery, Istanbul Faculty of Medicine, Istanbul University, Istanbul, Türkiye; ^2^ Department of Pathology, Istanbul Faculty of Medicine, Istanbul University, Istanbul, Türkiye; ^3^ Department of Pathology, Basaksehir Cam Sakura Hospital, Istanbul, Türkiye; ^4^ Multidisciplinary Oncologic Centre Antwerp (MOCA), Antwerp University Hospital, Edegem, Belgium; ^5^ Center for Oncological Research (CORE), University of Antwerp, Antwerp, Belgium; ^6^ Department of General Surgery, American Hospital, Istanbul, Türkiye; ^7^ Department of Medical Oncology, Institute of Oncology, Istanbul University, Istanbul, Türkiye; ^8^ Department of General Surgery, Istanbul Florence Nightingale Hospital, Istanbul, Türkiye

**Keywords:** CD155, CD73, chemotherapy response, prognosis, triple-negative breast cancer

## Abstract

**Background:**

Immune checkpoint inhibition, combined with novel biomarkers, may provide alternative pathways for treating chemotherapy-resistant triple-negative breast cancer (TNBC). This study investigates the expression of new immune checkpoint receptors, including CD155 and CD73, which play a role in T and natural killer (NK) cell activities, in patients with residual TNBC after neoadjuvant chemotherapy (NAC).

**Methods:**

The expression of biomarkers was immunohistochemically examined by staining archival tissue from surgical specimens (n = 53) using specific monoclonal antibodies for PD-L1, CD155, and CD73.

**Results:**

Of those, 59.2% (29/49) were found to be positive (>1%) for PD-L1 on the tumour and tumour-infiltrating lymphocytes (TILs), while CD155 (30/53, 56.6%) and CD73 (24/53, 45.3%) were detected on tumours. Tumour expressions of CD155 and CD73 significantly correlated with PD-L1 expression on the tumour (p = 0.004 for CD155, p = 0.001 for CD73). Patients with CD155 positivity ≥10% were more likely to have a poor chemotherapy response, as evidenced by higher MDACC Residual Cancer Burden Index scores and Class II/III than those without CD155 expression (100% vs 82.6%, p = 0.03). At a median follow-up time of 80 months (range, 24–239), patients with high CD73 expression showed improved 10-year disease-free survival (DFS) and disease-specific survival (DSS) rates compared to those with low CD73 expression. In contrast, patients with CD155 (≥10%) expression exhibited a decreasing trend in 10-year DFS and DSS compared to cases with lower expression, although statistical significance was not reached. However, patients with coexpression of CD155 (≥10%) and low CD73 were significantly more likely to have decreased 10-year DFS and DSS rates compared to others (p = 0.005).

**Conclusion:**

These results demonstrate high expression of CD73 and CD155 in patients with residual tumours following NAC. CD155 expression was associated with a poor response to NAC and poor prognosis in this chemotherapy-resistant TNBC cohort, supporting the use of additional immune checkpoint receptor inhibitor therapy. Interestingly, the interaction between CD155 and CD73 at lower levels resulted in a worse outcome than either marker alone, which calls for further investigation in future studies.

## Introduction

Triple-negative breast cancer (TNBC) is the most aggressive subtype, accounting for approximately 15–20% of all breast cancer cases (1). Recent studies in TNBC have indicated that high levels of stromal tumour-infiltrating lymphocytes (TILs) can serve as prognostic markers and may also predict patients’ responses to chemotherapy (2, 3). Clinical trials have demonstrated some efficacy of targeted therapy against programmed death ligand 1 (PD-L1)/programmed cell death 1 (PD-1) and have shown improved survival outcomes for TNBC patients (4–6). Consequently, the existing literature emphasizes the need for new immunotherapeutic approaches for TNBC. CD155 (7–10) and CD73 (11–14) are targetable molecules that could modulate the anti-tumour immune response and serve as potential promising prognostic biomarkers for clinical outcomes in breast cancer.

T cell immunoglobulin and ITIM domain (TIGIT) is a member of the CD28 protein family and has emerged as a new target for immunotherapy (15–19). It is predominantly expressed on T and natural killer (NK) cells and inhibits their anti-tumour activities. In the tumour microenvironment, T cells often co-express TIGIT along with other immune checkpoint receptors, such as PD-1 (20). CD155, a type I transmembrane glycoprotein, belongs to the immunoglobulin superfamily and serves as one of the ligands for TIGIT alongside low affinity nectin-2/CD112 and nectin-3/CD113 (21). Originally identified as a poliovirus receptor (PVR), CD155 is involved in various physiological processes, including cell proliferation, adhesion, and potentially tumour invasion and migration (22–25). CD155 is highly expressed on endothelial cells, dendritic cells, and fibroblasts, and its overexpression has been observed in several cancer types, such as lung adenocarcinoma, colorectal cancer, pancreatic cancer, cutaneous melanoma, and hepatocellular carcinoma (26–30). Notably, CD155 interacts with regulatory receptors CD96 and CD226 expressed on NK cells, CD4+ T cells, and monocytes. The CD155-CD226 interaction stimulates the cytotoxicity of NK cells and T cell response, while the CD155-CD96 interaction inhibits NK cell function (31). Any imbalance in this interaction may result in tumour immunosuppression (23). Given its role as an immune checkpoint protein, CD155 represents a potential target for novel anti-tumour immunotherapy in TNBC, with its overexpression serving as an indicator of poor prognosis (7).

CD73 is a GPI-anchored ecto-nucleotidase that is crucial in limiting the breakdown of extracellular ATP to adenosine (32, 33). Adenosine acts as an immunosuppressive molecule, inhibiting the activity of CD8+ T cells and NK cells while promoting the proliferation of immunosuppressive cells (34, 35). Within the tumour microenvironment, adenosine levels increase, leading to a reduction in the anti-tumour immune response by promoting the stabilization of immunosuppressive regulatory cells and suppressing the functions of effector cells (36). Thus, the CD73-adenosine pathway contributes to creating an immunosuppressive microenvironment in various tumours (37). Overexpression of CD73 has been observed in infiltrating immune cells and stromal tumour cells (38). Moreover, CD73 is upregulated on regulatory T cells in response to adenosine signalling and hypoxia (38–40). Recent studies have shown that CD73 expression may be a better predictor of neoadjuvant chemotherapy (NAC) response than TILs in TNBC (13).

The significance of CD155 and CD73 expressions on tumours in TNBC remains controversial. Additionally, the potential interaction between CD155 and CD73 is unknown, considering the complex immunoregulatory mechanisms involving TIGIT and CD155 and adenosine and CD73 in modulating T and NK cell responses. Therefore, this study aims to investigate the immunohistochemical expressions of CD155 and CD73, along with PD-L1 expression, and to analyze the associations between their expression levels, response to chemotherapy, and prognosis in TNBC patients.

## Materials and methods

Between September 2000 and May 2017, consecutive patients with TNBC diagnosed with locally advanced breast cancer, who underwent breast surgery at the Istanbul University, Istanbul Faculty of Medicine, Department of General Surgery, Breast Surgery Service after completing NAC, were included in the study. Patients with a pathologic complete response, male breast cancer, pregnancy-associated breast cancer, bilateral breast cancer, and distant metastases were excluded from the analysis. Patient and tumour characteristics were analyzed to evaluate the clinicopathological factors and outcomes in the study group. The American Joint Committee on Cancer Staging System 8th edition was used in clinical and pathological evaluation of patients (41). Ethical committee approval was obtained from the Istanbul University, Istanbul Faculty of Medicine.

### Immunohistochemical evaluation and scoring

Patients with TNBC were identified based on their previous pathology reports of the surgical specimen. All patients had negative estrogen and progesterone receptors and c-erb-B2 expressions, which were examined using immunochemistry (IHC). Immunological markers were retrospectively studied in archival tissue material of surgical specimens (n = 53) using immunohistochemistry. Tumour paraffin block sections containing TILs were chosen for immunostaining.

Immunohistochemical expressions of PD-L1, CD-73, and CD155 were detected using an automatic Ventana BenchMark slide staining device (Ventana Medical Systems, Tucson, AZ, USA). The 5-μm formalin-fixed paraffin-embedded sections were incubated with specific primary antibodies, including anti-CD73 rabbit mAb (D7F9A, Cell Signaling) at a 1:200 dilution, and anti-CD155 rabbit mAb (D8A5G, Cell Signaling) at a 1:200 dilution. PD-L1 expression was detected using the “rabbit monoclonal antibody, Ventana SP263 Clone kit” (Ventana Medical Systems, Tucson, AZ, USA). Placenta tissue was used as a control sample.

The staining percentage and intensity of tumour cells and TILs were recorded for each immune checkpoint receptor. The staining intensity was categorized as follows: no staining, weakly stained, moderately stained, or strongly stained. All immune checkpoint receptors, including PD-L1, CD73, and CD155, exhibited a membranous staining pattern. PD-L1 positivity was defined as membranous staining >1% on either tumour or TILs, or both, as previously described (42). Various staining percentages ranging from 1% to 20% (>1%, >5%, >10%, >20%), determined based on the median values for each biomarker, along with or without staining intensity, were tested to investigate significant associations with prognosis for CD73 and CD155. Furthermore, an expression score for CD73 and CD155 was calculated for each patient using the formula “staining intensity × percentage of positive cells” to evaluate its significance for the outcome. Stained tumour cells and TILs were assessed under a light microscope (Olympus BX51, Japan) at 40× magnification, equipped with an integrated digital camera (Olympus DP71, Japan).

The “MD Anderson Cancer Center Residual Cancer Burden Index” was calculated to assess the response to NAC based on the following residual tumour characteristics: a) The two largest dimensions of the residual tumour bed (including the largest tumour bed in multicentric cases), b) The histologic assessment of the percentage of the tumour bed area containing carcinoma, c) The histologic estimate of the percentage of carcinoma in the tumour bed that is *in-situ*, d) The number of metastatic lymph nodes, and e) The diameter of the largest lymph node metastasis. The “RCB” index was estimated using the MD Anderson Residual Cancer Calculator (www3.mdanderson.org/app/medcalc/index.cfm?pagename=jsconvert3) by incorporating these parameters. The residual cancer classification was determined based on this scoring system. A chemotherapy response was considered good if classified as Class 0 (pathologic complete response) or Class 1, and not as good if classified as Class 2 or 3 (chemotherapy resistant)

### Statistical analysis

The study’s statistical analysis was conducted using the SPSS 17 software program (Statistical Package for Social Sciences; SPSS, Inc, Chicago, IL). Categorical variables were assessed using the Pearson Chi-Square, Fisher’s exact, or Continuity Correction tests. Differences between continuous variables were evaluated using the Mann-Whitney U test. The Spearman correlation test examined the expression associations between continuous variables, including the percentages of CD155, CD73, and PD-L1. Disease-free survival (DFS) rates were analyzed, considering locoregional and distant recurrences, while disease-specific survival (DSS) rates were analyzed considering breast cancer-associated mortality. Kaplan-Meier analyses were performed to calculate DFS and DSS rates and construct survival curves. The log-rank test was used to compare factors influencing the outcome. A p-value less than 0.05 was considered statistically significant.

## Results

Of the 53 patients diagnosed with locally advanced TNBC, the mean age was 50 ± 13.3 (95% confidence interval (CI); 46.2–53.5), whereas the median age was 47 years (range, 24–76 years). Among them, 29 patients were clinically (= c) T3–4 (54.6%), while almost all of them had cN1-3 (96.2%) before NAC. All patients received NAC, including anthracyclines, followed by taxanes. Following completion of NAC, most patients (n = 39, 73.6%) underwent mastectomy and axillary dissection (n = 46, 86.8%). Breast-conserving surgery was performed in the remaining patients, and seven cases had only sentinel lymph node biopsy due to negative intraoperative pathological evaluation of the lymph nodes. In the definitive pathology evaluation of the surgical specimens, 16 cases (30.2%) showed axillary pathologic complete response (ypN0), while all patients had residual invasive cancer in the breast specimen. Histopathological examination revealed 43 tumours with invasive ductal carcinoma (81.1%), three tumours with invasive lobular carcinoma (5.7%), one tumour with mixed invasive ductal and lobular carcinoma (1.9%), and six tumours with metaplastic carcinoma (11.3%). The mean “MD Anderson Cancer Center Residual Cancer Burden Index” was 3.17 ± 1.2 (95%CI, 2.8–3.5).

### Staining patterns and associations with clinicopathological characteristics

**Figure 1:**
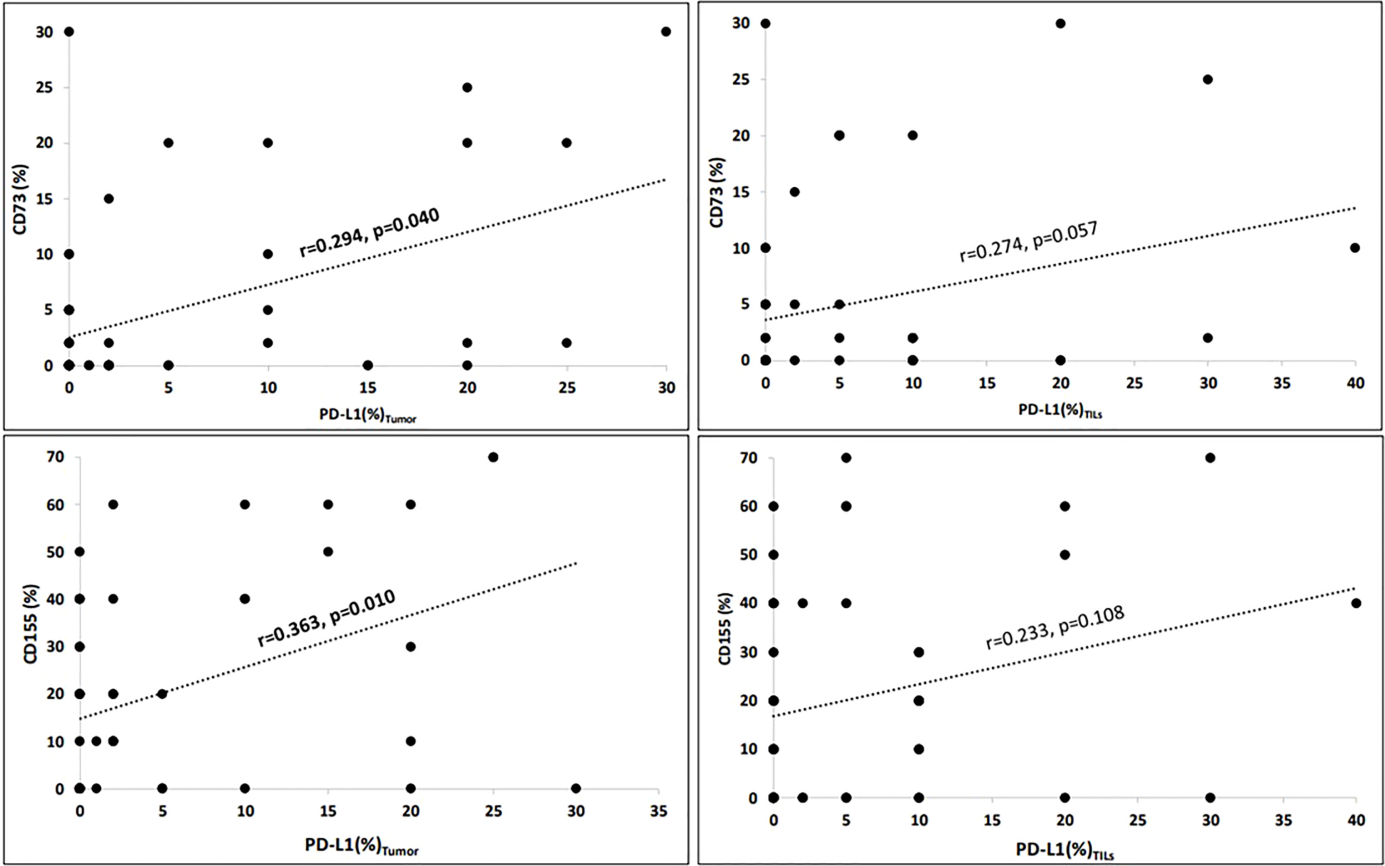
Correlations of immuncheckpoint receptors (Spearman’s rho). Tumour expressions of CD155 (%) and CD73 (%) significantly correlated with PD-L1_tumour_ (for CD73, r = 0.294, p = 0.040 and for CD155, r = 0.363, p = 0.010). However, the associations with PD-L1_TILs_ expressions did not reach the statistical significance (for CD73, r = 0.274, p = 0.057 and for CD155, r = 0.233, p = 0.108). Correlation is significant at the 0.05 level (2-tailed).

The mean values of PD-L1 expressions on tumours and TILs, as well as the expressions of CD73 and CD155 on the tumour (%), along with the CD73 and CD155 scores, are shown in **Table 1**. Tumour expressions of CD155 and CD73 were found to have a significant correlation with PD-L1_tumors_ (for CD73, r = 0.294, p = 0.040; and for CD155, r = 0.363, p = 0.010; **Figure 1**). However, the associations with PD-L1_TILs_ expressions did not reach statistical significance (for CD73, r = 0.274, p = 0.057; and for CD155, r = 0.233, p = 0.108).

**Table 1:** Immune check point expression levels.

Immune checkpoint receptor expression	Mean ± SD (95% Confidence Interval)
CD73 (%)	4.79 ± 8.22 (2.53-7.06)
CD73 score	8.17 ± 2.29 (3.59-12.76)
CD155 (%)	19.06 ± 3.05 (12.93-25.18)
CD155 score	28.11 ± 5.20 (17.67-38.55)
PD-L1_Tumour_ (%)	5.33 ± 8.31 (2.94-7.71)
PD-L1_TIL_ (%)	5.84 ± 9.12 (3.22-8.47)

**Figure 2:**
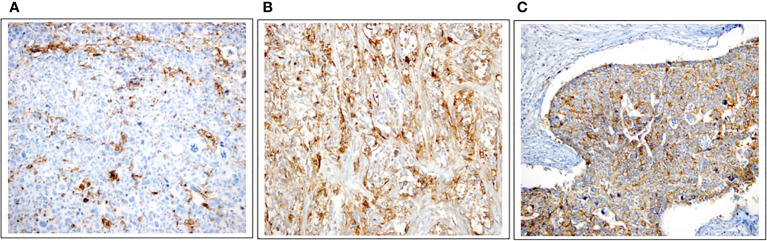
Immunohistochemical expressions of PD-L1, CD73, and CD155. **(A)** PD-L1 expression in the tumour with 25% strongly stained, in intratumoral lymphocytes with 5% -moderately stained (×20). **(B)** High expression of CD73 as membranous staining pattern on tumor cells (×20). **(C)** Positive CD155 expression (≥10%) as membranous staining pattern on tumor cells (×20).

PD-L1 expression was observed on tumours or TILs in 29 cases (59.2%, **Figure 2A**). Additionally, tumoural staining for CD73 was observed in 24 patients (45.3%, **Figure 2B**), while 30 patients exhibited tumoural CD155 expression (56.6%, **Figure 2C**).

Low CD73 expression was considered if the tumour cells were weakly stained <20%. High CD73 expression was considered if the tumour cells were weakly stained ≥20% or any moderately/strongly staining. Patients with high CD73 expression (n = 11, 20.8%) were observed to have a higher likelihood of achieving an axillary pathologic complete response compared to those with low CD73 expression (54.6% vs 23.8%, p = 0.068); however, this difference did not reach statistical significance. In contrast, patients expressing CD155 were more likely to exhibit a poor chemotherapy response, as indicated by higher MD Anderson Cancer Center Residual Cancer Burden Index scores and Class II/III, compared to those without CD155 expression (100% vs 82.6%, p = 0.03; **Table 2**). Nevertheless, no significant associations were found between CD73 and CD155 expressions and other clinicopathological characteristics. Furthermore, no significant associations could be found in CD73 high-expression (n=11) among patients with CD155 ≥10% vs CD155 <10% expression (5/30, 16.7% vs 6/23, 26.1%, p=0.501, respectively). Patients with CD155 ≥10% were more likely to exhibit PD-L1_total_ positivity compared to others (21/30, 70% vs. 8/19, 42.1%, p = 0.05, respectively). Similarly, patients with high CD73 expression were more likely to have PD-L1_total_ positivity than those with low CD73 expression (9/10, 90% vs 20/39, 51.3%, p = 0.034, respectively).

**Table 2:** Associations of immune checkpoint receptor expression with clinicopathological factors.

Variables		CD73		*p-value*	CD155		*p-value*
	All (n = 53)	Low (n = 42)	High (n = 11)		<10% (n = 23)	≥10% (n = 30)	
	n	n(%)	n(%)		n(%)	n(%)	
Age				*0.735^a^*			*0.546^b^*
≤ 50	29 (54.7%)	22(52.4)	7(63.6)		11(47.8)	18(60)	
>50	24 (45.3%)	20(47.6)	4(36.4)		12(52.2)	12(40)	
				*0.518^a^*			*0.962^b^*
cT1-2	24 (45.3%)	18(42.9)	6(54.5)		11(47.8)	13(43.3)	
cT3-4	29 (54.7%)	24(57.1)	5(45.5)		12(52.2)	17(56.7)	
				*0.999^a^*			*0.639^b^*
cN0-1	33 (62.3%)	26(61.9)	7(63.6)		13(56.5)	20(66.7)	
cN2-3	20 (37.7%)	16(38.1)	4(36.4)		10(43.5)	10(33.3)	
				*0.068^a^*			*0.737^b^*
ypN0	16 (30.2%)	10(23.8)	6(54.5)		8(34.8)	8(26.7)	
ypN(+)	37 (69.8%)	32(76.2)	5(45.5)		15(65.2)	22(73.3)	
MDACC RCBI				*0.624*			0.028^c*^
Mean Score ± SD (95%CI)	3.2 ± 1.2 (2.8-3.5)	3.2 ± 1.2 (2.8-3.6)	3 ± 1.1 (2.2-3.7)		2.8 ± 1.3 (2.2-3.3)	3.5 ± 1.1 (1.6-5.1)	
MDACC RCBI				*0.569^a^*			0.030^a*^
Class I	4 (7.5%)	4(9.5)	0(0)		4(17.4)	0(0)	
Class II-III	49 (92.5%)	38(90.5)	11(100)		19(82.6)	30(100)	
				*0.313^a^*			*0.141^b^*
Class I-II	25 (47.2%)	18(42.9)	7(63.6)		14(60.9)	11(36.7)	
Class III	28 (52.8%)	24(57.1)	4(36.4)		9(39.1)	19(63.3)	

MDACC RCBI, MDACC Residual Cancer Burden Index.

*p<0.05, Chi-Square Tests (^a^Fisher’s Exact Test, ^b^Continuity Correction), ^c^Mann Whitney U test

cT: clinical T size (determined by physical exam and imaging, AJCC 8^th^ edition) (42);

cN: clinical nodal status (determined by physical exam and imaging, AJCC 8^th^ edition) (42);

ypN0: pathological nodal complete response after neoadjuvan chemotherapy (AJCC 8^th^ edition) (42);

ypN(+): pathological residual nodal disease after neoadjuvant chemotherapy (AJCC 8^th^ edition) (42).

Low CD73 expression was considered if the tumour cells were weakly stained <20%. High CD73 expression was considered if the tumour cells were weakly stained ≥20% or any moderately/strongly staining.

### Outcome

**Figure 3:**
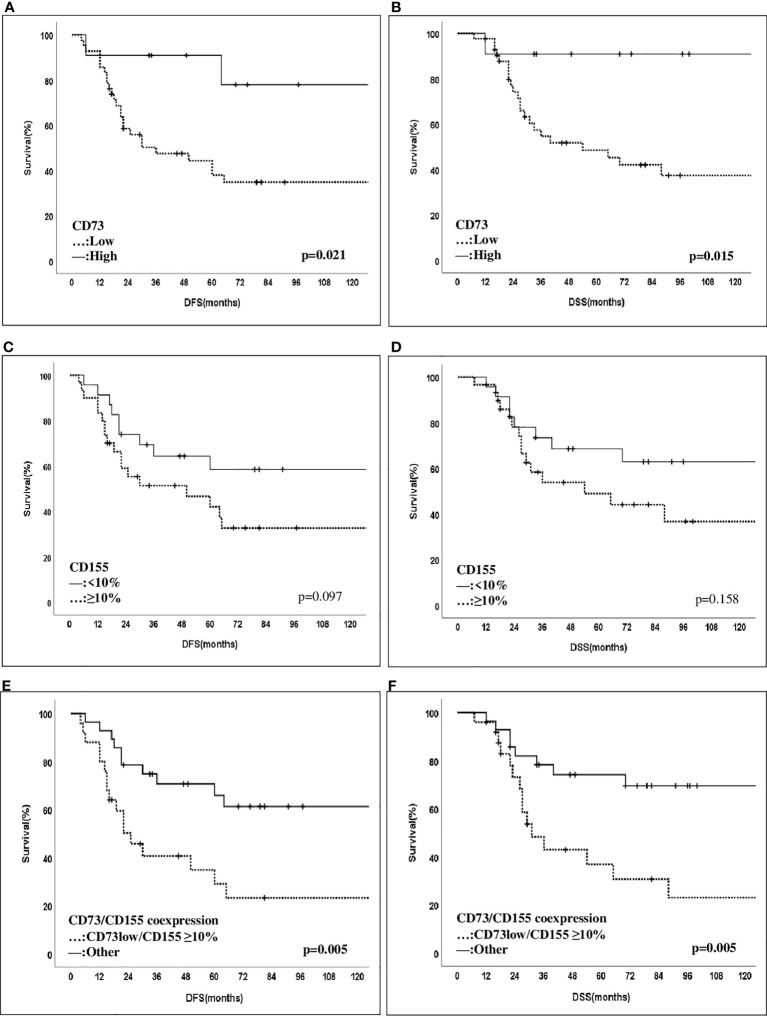
Disease-free and disease-spesific survival of patients with CD73 and CD155 expressions. Patients with CD73-high expression were found to have an improved 10-year DFS- and DSS rate compared to those with CD73-low expression (10-year DFS: 34.8% vs 77.9%, p = 0.021, and 10-year DSS: 37.3% vs 90.9%, p = 0.015) **(A, B)**. Those with a ≥10% CD155 expression have contrastly shown a decreased trend of 10-year-DFS and DSS compared to other cases with lower expression patterns (10-year DFS: 32.7% vs 58.3%, p = 0.097, and 10-year DSS: 36.7% vs 62.8%, p = 0.158) **(C, D)**. Notably, patients with coexpression of CD155 (>10%)/CD73-low were significantly more likely to have a decreased 10-year DFS and DSS rate compared to others (p = 0.005) **(E, F)**.

The median follow-up time was 80 months (range, 24–239 months). In univariate survival analyses (**Figure 3**), patients with high CD73 expression showed an improved 10-year DFS and DSS rate compared to those with low CD73 expression. On the other hand, patients with CD155 expression (≥10%) demonstrated a decreasing trend in 10-year DFS and DSS rates, although it did not reach statistical significance. Notably, patients with coexpression of CD155 (≥10%)/CD73-low were significantly more likely to have a decreased 10-year DFS and DSS rate compared to others (p = 0.005). However, no other significant associations were found between the expression patterns of CD73, CD155, PD-L1, CD73PD-L1, or CD155PD-L1 and outcomes (**Table 3**). Furthermore, in multivariate Cox regression analysis, patients with a higher MD Anderson Cancer Center Residual Cancer Burden Index (RCBI) had an increased hazard ratio (HR) of DFS (HR = 1.941; 0.838–4.495) and DSS (HR = 2.904; 1.103–7.643) compared to those with better chemotherapy response. It is worth noting that patients with low CD73 expression had a higher HR of DFS (HR = 3.979; 0.926–17.102) and DSS (HR = 6.45; 0.858–48.490) compared to those with high CD73 expression, although statistical significance was not reached (**Table 4**).

**Table 3:** Outcome of patients according to biomarker expressions with different cut-off levels and staining patterns.

Biomarker expression	N (%)	10-year DFS (%)	*p-value*	10-year DSS (%)	*p-value*
CD73 (%, n = 53)					
CD73			0.303		0.490
<1%	29 (54.7%)	37.1		41.6	
≥1%	24 (45.3%)	54.0		59.9	
CD73			0.548		0.576
<5%	36 (67.9%)	40.2		43.4%	
≥5%	17 (32.1%)	52.8		62.7%	
CD73			0.475		0.324
<10%	42 (79.2%)	40.7		43.4%	
≥10%	11 (20.8%)	58.2		70.7%	
CD73			0.179		0.099
<20%	46 (86.8%)	39.8		42.0%	
≥20%	7 (13.2%)	68.6		85.7%	
CD73 expression*			0.021*		0.015*
Low (weakly stained <20%)	42 (79.2%)	34.8		37.3	
High (moderately/strongly staining &weakly stained if ≥20%)	11 (20.8%)	77.9		90.9	
CD73 score (n = 53)					
Score			0.548		0.576
<5	36 (67.9%)	40.2		43.4	
≥ 5	17 (32.1%)	52.8		62.7	
Score			0.293		0.199
<10	41 (77.4%)	39.0		41.5	
≥ 10	12 (22.6%)	62.5		73.3	
Score			0.123		0.070
<20	45 (84.9%)	39.1		41.3	
≥ 20	8 (15.1%)	70.0		87.5	
CD155 (%, n = 53)			0.097		0.158
<10%	23 (43.4%)	58.5		62.8	
≥10%	30 (56.6%)	32.7		36.7	
CD155			0.115		0.285
<20%	24 (46.1%)	56.4		59.7	
≥20%	28 (53.9%)	28.9		32.2	
CD155			0.218		0.446
<30%	30 (57.7%)	50.2		53.5	
≥30%	22 (42.3%)	31.4		41.0	
CD155 score (n = 53)					
Score			0.097		0.158
<10	23 (43.4%)	58.5		62.8	
≥ 10	30 (56.6%)	32.7		36.7	
Score			0.115		0.285
<20	29 (54.7%)	56.4		59.7	
≥ 20	24 (45.3%)	28.9		32.2	
Score			0.141		0.424
<40	32 (60.4%)	51.2		53.2	
≥ 40	21 (39.6%)	33.0		42.8	
Score			0.767		0.893
<50	40 (75.5%)	42.8		49.5	
≥ 50	13 (24.5%)	46.2		48.6	
PD-L1 (%)					
Tumour			0.687		0.878
–	24 (49%)	44.6		48.5	
+	25 (51%)	39.0		45.9	
TILs			0.405		0.255
–	24 (49%)	34.5		37.5	
+	25 (51%)	48.9		55.1	
Total			0.822		0.858
–	20 (40.8%)	43.1		47.6	
+	29 (59.2%)	41.2		46.6	
CD73/CD155 coexpression			0.005		0.005
CD73low/CD155 ≥10%	25 (47.2%)	23.2		23.0	
^a^Other (n = 28)	28 (52.8%)	61.2		69.5	
CD73/PD-L1_Total_ coexpression			0.072		0.046*
CD73high/PD-L1_Total_ (+)	9 (17.3%)	71.1		88.9	
^b^Other	43 (82.7%)	36.6		39.3	
CD155/PD-L1_Total_ coexpression			0.289		0.209
CD155(≥10%)/PD-L1_Total_ (+)	21 (39.6%)	34.9		36.3	
^c^Other	32 (60.4%)	50.9		58.0	

*: p<0.05; χ^2^: Log-Rank (Mantel-Cox)

a Other: CD73high/PD-L1_Total_ (-), CD73low/PD-L1_Total_ (-), CD73low/PD-L1_Total_ (+)

b Other: CD73high/PD-L1_Total_ (-), CD73low/PD-L1_Total_ (-), CD73low/PD-L1_Total_ (+)

c Other: CD155(≥10%)/PD-L1_Total_ (-), CD155(-)/PD-L1_Total_ (-), CD155(-)/PD-L1_Total_ (+).

**Table 4:** Multivariate cox regression analysis.

Factors	Disease-free Survival	*p-value*	Disease-specific Survival	*p-value*
	HR (95%CI)		HR (95%CI)	
MDACC Residual Cancer Burden Index		0.122		0.031
Class I-II	Reference (1)		Reference (1)	
Class III	1.941 (0.838-4.495)		2.904(1.103-7.643)	
CD73		0.063		0.070
high (weakly stained <20%)	Reference (1)		Reference (1)	
low (moderately/strongly staining & weakly stained if ≥20%)	3.979(0.926-17.102)		6.451 (0.858-48.490)	
CD155		0.246		0.453
<10	Reference (1)		Reference (1)	
≥10%	1.636 (0.712-3.758)		1.407(0.577-3.430)	

Hazard ratio (HR) are presented with their 95% confidence interval (CI) and the p-value.

## Discussion

There are currently no established molecular targets for TNBC patients, so chemotherapy remains the standard treatment approach. However, unlike patients with other subtypes, TNBC patients typically exhibit aggressive clinical behaviour and have an unfavourable prognosis. Consequently, novel systemic therapies, including immunotherapies, are being investigated for TNBC patients who are resistant to neoadjuvant chemotherapy or have only achieved a partial response to NAC. CD73 and CD155 have recently garnered significant attention as potential therapeutic targets for their immunoregulatory functions (18, 19, 21, 22, 43–45).

CD155 has emerged as a novel immune checkpoint protein highly expressed in many tumour cells (26–30). Its expression has been implicated in tumour immunosuppression (3), as its interaction with TIGIT or CD96-positive T lymphocytes and NK cells leads to immune exhaustion and reduced interferon-γ secretion (4, 5). Therefore, blocking CD155-TIGIT or CD96 signalling could enhance anti-tumour immune cell function, making it a potential marker for immunotherapy in breast cancer (43–45).

CD73, also known as ecto-5′-nucleotidase (NT5E), is the rate-limiting enzyme in the ATP to adenosine degradation pathway. It regulates the synthesis of adenosine through the catabolism of extracellular ATP (1, 2). Growing evidence suggests that the CD73-adenosine pathway plays a critical role in cancer progression and immune surveillance, exerting immunosuppressive effects on NK cells and CD8+ T cells, which can stimulate tumour escape mechanisms. Therefore, we investigated the potential interaction between these novel immune checkpoint expressions in response to NAC and the prognosis of patients with residual TNBC.

Our study found that CD155 was associated with poor chemotherapy response and outcome, whereas CD73 overexpression was conversely indicative of improved survival. Intriguingly, the interaction of CD155 with CD73 at lower levels resulted in a worse outcome than either protein alone. Furthermore, both CD73 and CD155 were found to be associated with PD-L1 expression in TNBC within our cohort.

There have been limited studies investigating the prognostic significance of CD155 immunohistochemical expression (IHC) in breast cancer (7, 10, 46, 47). In a study conducted by Yoshikawa et al. (7), CD155 expression was observed in 41% (25/61) of TNBC patients using IHC and tissue microarray. However, no associations were found between CD155 expression and pathological stage, histological grade, Ki-67 labelling index, or stromal tumour-infiltrating lymphocytes. Notably, only PD-L1 expression in tumour cells, as determined by the SP142 assay, exhibited a significant correlation with CD155 expression (p = 0.035). Our present study also found correlations between CD155 expression on tumour cells and PD-L1 expression on both tumour cells and tumour-infiltrating lymphocytes. However, unlike the current cohort, Yoshikawa et al. found no significant associations between CD155 expression and DFS or overall survival (OS).

Yong et al. conducted a study involving 216 patients and similarly found a significant association between CD155 expression, as determined by IHC, and primary tumour size, lymph node metastasis, TNM stage, Ki-67 expression, and CD163/CD8/CD68 expression (10). Among the cases, 117 had ER-negative tumours, and nearly half had HER2-positive cancer. Most of the cohort consisted of early-stage breast cancer patients who underwent upfront surgery. Importantly, patients with high CD155 expression were more likely to experience poor OS, as indicated by both univariate analysis (HR = 2.681, 95%CI = 1.458–4.928, p < 0.001) and multivariate analysis (HR = 2.029, 95%CI = 1.059–3.887, P = 0.033). Consistent with our findings, multivariate analysis further confirmed that CD155 expression level and TNM stage were independent risk factors for OS. These findings suggest an interaction between CD155 expression and TILs in breast cancer and highlight the potential utility of CD155 as a prognostic marker.

In a recent study conducted by Li et al. (46), CD155 overexpression was detected in 17%, 39%, 37%, and 62% of patients diagnosed with Luminal A, Luminal B, HER2-positive, and TNBC, respectively, in a cohort of 126 patients. Patients with CD155 overexpression exhibited a higher Ki-67 index and a greater presence of tumour-infiltrating lymphocytes and PD-1+ lymphocytes than those with low expression. Additionally, patients with CD155 overexpression experienced significantly poorer DFS and OS (p < 0.05), along with an increased risk of recurrence (HR = 13.93, 95%CI: 2.82, 68.91) and death (HR = 5.47, 95%CI: 1.42–20.9), consistent with the findings of our present study.

A recent meta-analysis (47) involving 26 studies and 4,325 cancer patients revealed that high CD155 expression was significantly associated with decreased OS compared to low CD155 expression (pooled HR = 1.772, 95%CI = 1.441–2.178, p < 0.001). Moreover, a subgroup analysis specifically focusing on breast cancer patients demonstrated a significant association between CD155 expression and decreased OS (pooled HR = 2.137, 95%CI = 1.448–3.154, p < 0.001). Consistent with previous studies (7), we observed a high expression of CD155 in 57% of TNBC patients within our cohort. Interestingly, in our cohort of patients with residual breast cancer after NAC, those with high CD155 expression were more likely to respond poorly to NAC. These findings, combined with our present report, suggest that CD155 may serve as a potential target for immunotherapy in breast cancer.

Moreover, our study revealed that more than half of the patients (59%) exhibited PD-L1 expression on both tumour cells and TILs, while CD73 expression on tumour cells was observed in 45% of the patients. In contrast to the findings of the study by Buisseret et al. (48), our study demonstrated correlations between CD73 expression on tumour cells and PD-L1 expression on both tumour cells and TILs. However, in our cohort of patients with residual tumours following NAC, no significant associations were found between CD73 expression and the response to NAC. Nevertheless, Cerbelli et al. demonstrated a higher likelihood of achieving a pathological complete response (pCR) in a cohort of 61 TNBC patients with low CD73 expression as determined by immunohistochemical staining (13).

Controversial findings have emerged regarding the prognostic significance of CD73 expression in breast cancer (11–14). Loi et al. analyzed gene expression data from over 6,000 TNBC patients and determined that CD73 expression was associated with poor prognosis (12). Additionally, high CD73 gene expression was significantly correlated with a lower rate of pathological complete response in TNBC patients treated with anthracycline-only preoperative chemotherapy. In *in vitro* assays utilizing breast cancer cell lines, it was demonstrated that doxorubicin treatment increased CD73 expression in tumour cells, potentially leading to chemoresistance in mouse models. However, blocking CD73 resulted in enhanced anti-tumour immune responses to doxorubicin and prolonged the survival of mice in an established metastatic mouse model.

A recent meta-analysis encompassing 2,951 patients from 14 publications explored the associations between CD73 expression, clinicopathological characteristics, and prognosis across different cancers (14). The analysis revealed that high CD73 expression was significantly associated with decreased OS in breast cancer (HR = 1.23) and ovarian cancer (HR = 1.14), while it correlated with favourable OS in lung cancer (HR = 0.80) and gastric cancer (HR = 0.71). High CD73 expression was also strongly linked to lymph node metastases (OR = 2.61, p = 0.05). Our study found that patients with high CD73 expression were more likely to achieve axillary pathologic complete response than those with low CD73 expression (54.6% vs 23.8%, p = 0.068); however, this difference did not reach statistical significance.

In contrast to studies reporting CD73 as a poor prognostic indicator, our findings revealed an intriguing observation. We demonstrated an improved 10-year DFS and DSS rate in patients with high CD73 expression, as determined by immunohistochemistry (IHC), compared to those with low CD73 expression. These results were obtained at a median follow-up time of 80 months. Interestingly, our findings align with a report by Supernat et al., which indicated that CD73 expression, as assessed by IHC on tissue microarrays, serves as a favourable prognostic marker in 136 stage I-III breast cancer patients (11).

Furthermore, we present a novel finding in this study: the interaction between CD155 and CD73 at lower expression levels resulted in a worse outcome than either protein alone. This observation warrants further investigation in future studies. Consequently, the precise role of CD73 and its interaction with CD155 in cancer progression remains unclear and should be elucidated through *in vitro* and clinical studies.

## Conclusions

There is a critical need for novel targets in anti-cancer immunotherapy to improve the prognosis of TNBC patients. In this study, we demonstrated high expression of CD73 and CD155 in patients who had a partial response to NAC. Notably, CD155 expression was associated with a poor response to NAC and an unfavourable prognosis in this cohort of patients with residual TNBC, suggesting the potential benefit of additional immune checkpoint receptor inhibitor therapy. Consistent with other published studies (49–52), our findings also support the hypothesis that CD73 and CD155 could serve as promising therapeutic targets in TNBC, either alone or in combination with other immunotherapeutic agents targeting PD-L1. This opens avenues for developing personalized de-escalation or escalation strategies in patients with residual TNBC.

## Data availability statement

The original contributions presented in the study are included in the article/supplementary material. Further inquiries can be directed to the corresponding author.

## Ethics statement

The studies involving human participants were reviewed and approved by Istanbul University, Istanbul Faculty of Medicine, Ethical Committee. Written informed consent for participation was not required for this study in accordance with the national legislation and the institutional requirements.

## Author contributions

The study was designed by NC. The initial search, literature organization, and analyses were performed by NC, SO, HK, GO and EY. Manuscript writing was performed by NC, AB, SE, SO and GO. Critical comments and typesetting corrections on the final version were made by MT, MM, AI, AA, PS, EY and VO. The manuscript was finalized by NC. All authors have read and revised the manuscript critically. All authors contributed to the article and approved the submitted version.
